# Prognostic significance of systemic inflammatory response markers in patients with superficial esophageal squamous cell carcinomas

**DOI:** 10.1038/s41598-022-21974-y

**Published:** 2022-10-29

**Authors:** Chi-Jen Chen, Ching-Tai Lee, Ying-Nan Tsai, Chao-Ming Tseng, Tzu-Haw Chen, Ming-Hung Hsu, Chih-Chun Wang, Wen-Lun Wang

**Affiliations:** 1 Department of Internal Medicine, E-Da Hospital/I-Shou University, No. 1, Yida Road, Jiaosu Village, Yanchao District, Kaohsiung City, 82445 Taiwan; 2 Department of Internal Medicine, E-Da Cancer Hospital, Kaohsiung, Taiwan; 3 Department of Otolaryngology, E-Da Hospital/I-Shou University, Kaohsiung, Taiwan; 4 School of Medicine, College of Medicine, I-Shou University, Kaohsiung, Taiwan

**Keywords:** Cancer, Immunology, Biomarkers, Gastroenterology, Oncology, Risk factors

## Abstract

Endoscopic resection or esophagectomy has becoming the standard treatment for superficial esophageal squamous cell carcinomas (SESCC), but some patients may develop disease progression or second primary cancers after the therapies. Neutrophil to lymphocyte ratio (NLR), lymphocyte to monocyte ratio (LMR), and platelet to lymphocyte ratio (PLR) reflect the balance between pro-cancer inflammatory and anti-cancer immune responses, however their roles in SESCC are still unknown. We consecutively enrolled patients with newly diagnosed SESCC (clinical stage Tis or T1N0M0) who were treated at our institute. Pre-treatment NLR, LMR and PLR were assessed and then correlated with clinical factors and long-term survival. A total of 156 patients were enrolled (152 males, 4 females; median age: 52.2 years), of whom 104 received endoscopic resection and 52 were treated with esophagectomy or chemoradiation.. During a mean follow-up period of 60.1 months, seventeen patients died of ESCCs, and 45 died of second primary cancers. The 5-year ESCC-specific survival and 5-year overall survival rate were 86% and 57%, respectively. LMR (*P* < 0.05) and NLR (*P* < 0.05), but not PLR were significantly correlated with overall survival. Receiver operating characteristic curve analysis showed optimal LMR and NLR cut-off values of 4 and 2.5, respectively, to predict a poor prognosis. Patients with a high NLR or low LMR tended to have longer tumor length, larger circumferential extension, and presence of second primary cancers. Multivariate Cox regression analysis showed that presence of second primary cancers (HR: 5.05, 95%CI: 2.75–9.28), low LMR (HR: 2.56, 95%CI: 1.09–6.03) were independent risk factors for poor survival. A low pre-treatment LMR may be a non-invasive pretreatment predictor of poor prognosis to guide the surveillance program, suggesting that anti-cancer immunity may play a role in the early events of esophageal squamous cancer.

## Introduction

Esophageal cancer is the eighth most common cancer and the sixth most common cause of cancer death worldwide^1–3^. The incidence is highest in Asia, with up to 95% being reported in China^4^. The prognosis of patients with esophageal neoplasia is still extremely poor, even if they receive aggressive surgery or chemoradiation therapy. Recently, image-enhanced endoscopy techniques such as Lugol chromoendoscopy and narrow-band imaging have been shown to improve the detection and diagnosis of superficial esophageal squamous cell carcinoma (SESCC)^5,6^. In addition, endoscopic resection (endoscopic mucosal resection/endoscopic submucosal dissection) and esophagectomy are the standard treatments for SESCC^7^. A recent meta-analysis revealed that TP53, CCND1, and MDM2 are the most prevalent gene mutations, which can highlight their role in the carcinogenesis of ESCC^8^. Nevertheless, some cases may develop recurrence, metastasis or second primary cancers (e.g. Head and neck cancers) after the standard treatment^9,10^. Previous studies showed the multiple small lugol-voiding lesions in the esophageal background mucosa, presence of poor histological features (e.g. deep submucosal invasion, lymphovascular invasion) in the resected specimens are associated with a worse outcome^7,11–15^. However, a pre-treatment non-invasive biomarker to predict the long-term outcome of SESCC has yet to be identified.

Several recent studies have demonstrated that systemic inflammation and anti-tumor immunity play crucial roles in carcinogenesis, treatment effect, and long-term outcomes^16^. Increasing evidence has demonstrated that neutrophil to lymphocyte ratio (NLR), lymphocyte to monocyte ratio (LMR), and platelet to lymphocyte ratio (PLR) reflect the balance between pro-cancer inflammatory and anti-cancer immune responses, and they have been associated with the prognosis of gastroesophageal tract cancers^17–21^.

Most previous studies have reported the roles of NLR/LMR/PLR in patients with advanced-stage esophageal squamous cell carcinoma (ESCC) or in those who received chemoradiation therapy^21–24^, however studies focusing on superficial esophageal cancer or endoscopic resection are still lacking. Therefore, the aims of this study were to evaluate the associations between NLR, LMR, PLR and SESCC, and identify a non-invasive convenient biomarker for the long-term prognosis of patients with SESCC. The realization of the prognostic significance of these systemic inflammatory response markers in patients with SESCC may not only guide the surveillance program after treatment, but also provide the potential targets for prevention of esophageal cancers.

## Materials and methods

### Patients and design

We consecutively enrolled patients with newly diagnosed SESCC (clinical stage Tis or T1N0M0) at E-Da Hospital from January 2008 to October 2018. All of the included patients received treatment and follow-up at our institute, and pre-treatment complete blood cell count and differential count data were available. All patients underwent computed tomography (CT) to confirm that there was no lymph node involvement or distant metastasis. The baseline demographic and endoscopic characteristics, and data on alcohol drinking, betel nut chewing, and cigarette smoking were extracted from medical records. Treatment was performed based on the National Comprehensive Cancer Network (NCCN) clinical guidelines^25^. After treatment, the pathological stages, cancer invasion depth and the status of lymphovascular invasion were recorded based on the histopathological evaluation. The date of last follow-up or death was ascertained from medical records or by telephone contact. The data of cause of death and whether the patients presented with secondary primary cancer^10,12^, which indicated a second cancer develop on another site, such as head & neck or lung, were extracted from medical records. Informed consent was obtained from all of the patients. The study protocol was approved by the Institutional Review Board of E-Da Hospital (EMRP39101N) and conformed to the Declaration of Helsinki and Good Clinical Practice guidelines.

### Measurement of pre-treatment NLR, LMR and PLR

The pretreatment hematological parameters, including neutrophil count, lymphocyte count, monocyte count and platelet count were collected within 2 weeks before the initial treatment. The NLR, PLR and LMR were calculated using the absolute values of the corresponding hematological parameters, and they were then correlated with clinical features and long-term survival.

### Statistical analysis

All statistical analyses were performed using SPSS software (SPSS for Windows, version 22.0; SPSS Inc., Chicago, IL, USA). Comparisons between the different treatment groups and the clinical characteristics were performed using the χ^2^ test or the t-test as appropriate. The cumulative cancer-related survival rates were estimated using Kaplan–Meier curves and assessed using the log-rank test. Cox proportional hazard analysis was used to assess the factors associated with a worse outcome. A *p* value less than 0.05 was considered to indicate a statistically significant difference.

## Results

### Patient and endoscopic characteristics

A total of 156 patients (152 males and 4 females) with SESCC were included in this study, including 59 with high-grade intraepithelial neoplasia and 97 with T1 squamous cell carcinoma. The median age was 52.2 years, and more than 90% of the patients drank alcohol and/or smoked cigarettes. The mean tumor size was 3.6 cm. Of the enrolled patients, 104 were treated with endoscopic resection and 52 were treated with esophagectomy or chemoradiation. A total of 68 patients were found to have second primary cancers during the follow-up period, including 61 head & neck cancers, 3 gastric cancers, 2 lung cancers and 2 unknown primaries. The demographic and clinical data of the patients are shown in Table 1.

**Table 1:** Demographic and clinical features of the patients with ESCC.

Factors	Number (%)
Age, years, mean ± SD	52.2 ± 9.0
Sex, Female	4 (2.5%)
**Body mass index (BMI)**	20.9 ± 2.8
Alcohol drinking	146 (94%)
Betel nut chewing	102 (65%)
Cigarette smoking	147 (94%)
Tumor length, mean ± SD, cm	3.6 ± 2.6
**SESCC location**	
Upper	24 (15%)
Middle	86 (55%)
Lower	46 (29%)
**Second primary cancer**	
Head & neck cancer	61 (39%)
Gastric cancer	3 (1.9%)
Lung cancer	2 (1.3%)
Unknown primary	2 (1.3%)
**Absolute cell counts**	
Neutrophils (× 10^3^)	4.8 ± 3.0
Lymphocytes (× 10^3^)	1.4 ± 0.8
Monocytes (× 10^3^)	0.5 ± 0.2
Platelets (× 10^3^)	221.4 ± 92.1
**Ratios**	
NLR	6.4 ± 11.9
LMR	3.5 ± 2.3
PLR	14.7 ± 15.6

### NLR, LMR and PLR and their correlations with clinical features

**Figure 1:**
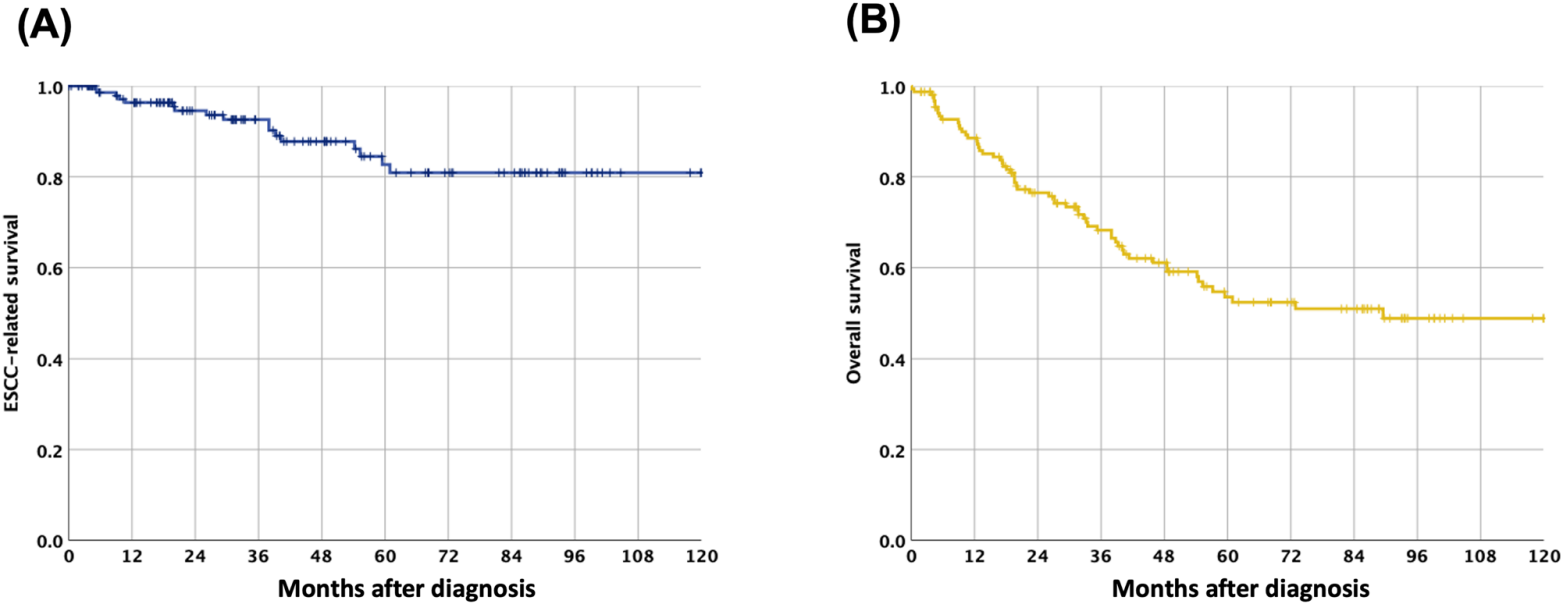
Kaplan–Meier survival curves showing ESCC-related survival (**A**) and the overall survival (**B**).

**Figure 2:**
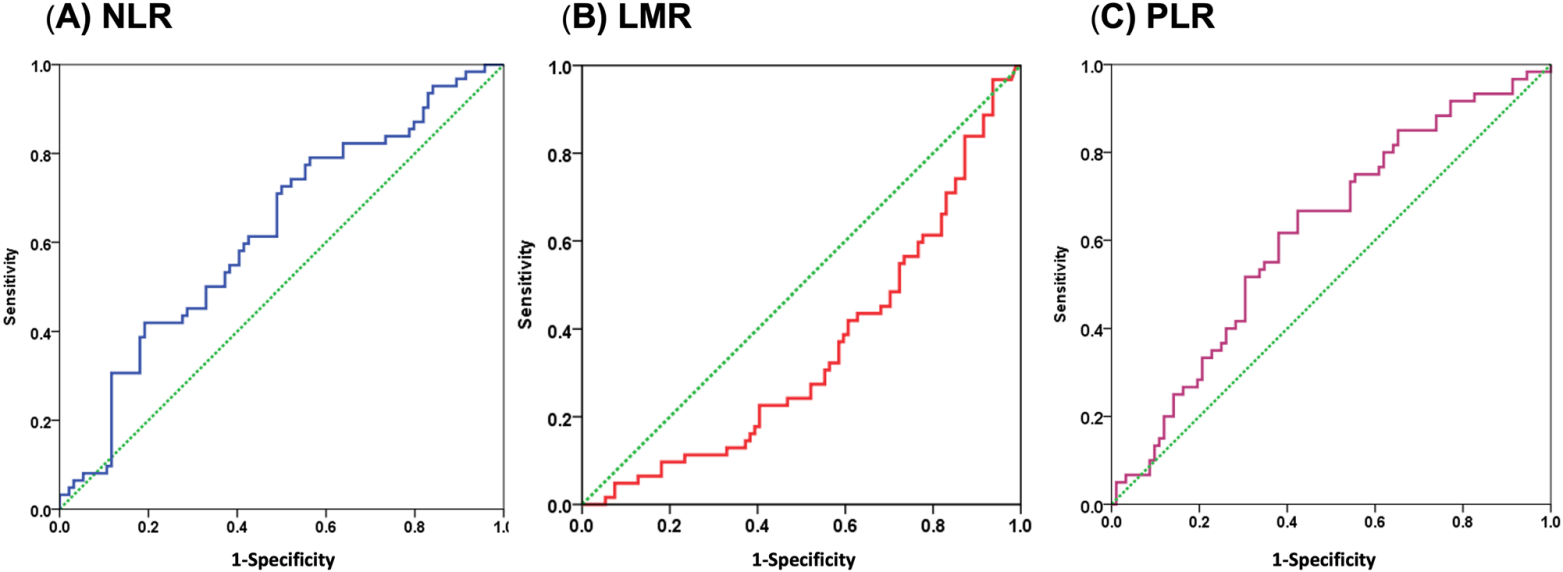
The optimal cut-off values were 2.5 for NLR (**A**), 4.0 for LMR (**B**), and 5.0 for PLR (**C**) in ROC curve analysis.

**Figure 3:**
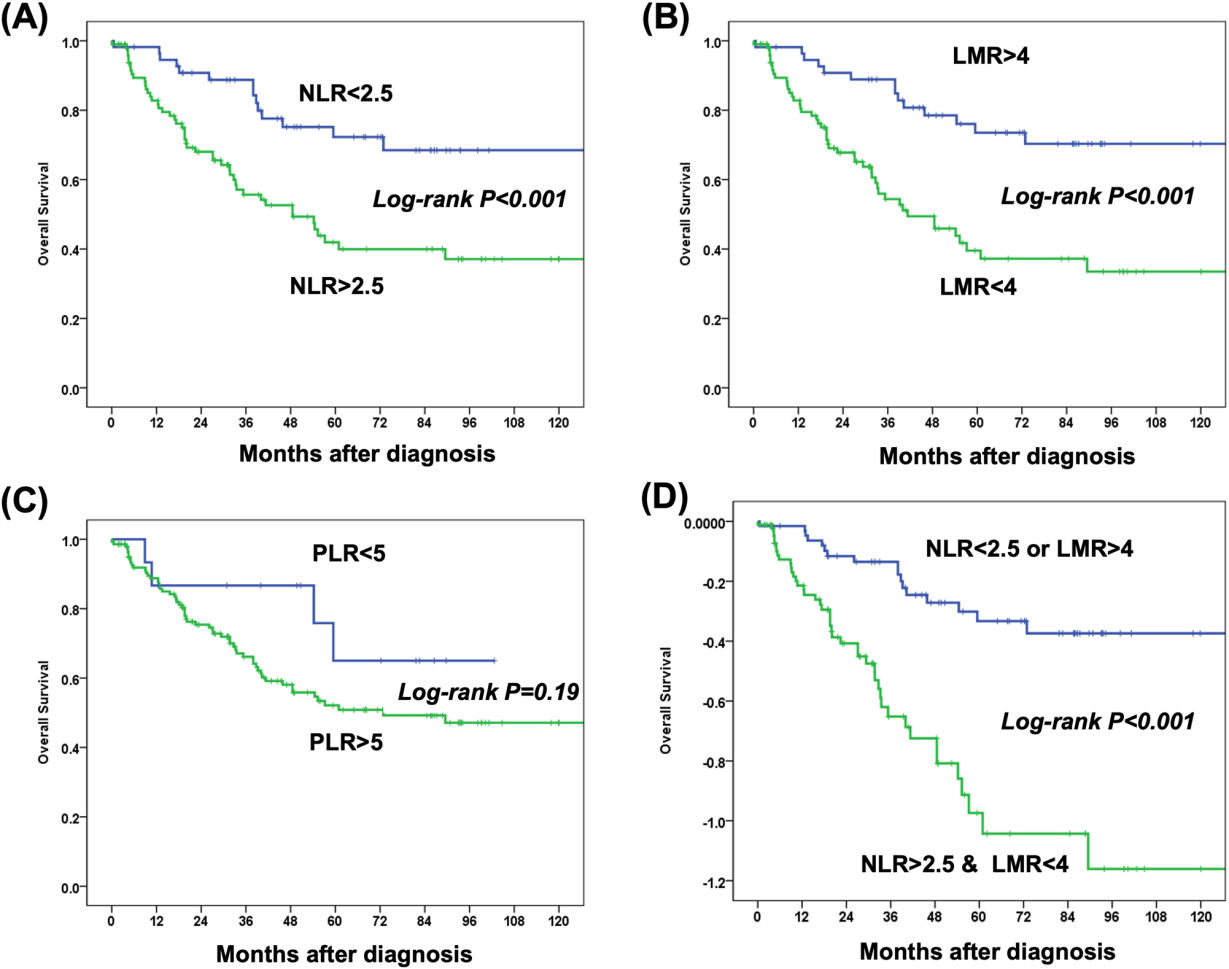
Kaplan–Meier survival curves showing the patients with a high NLRs (**A**), low LMR (**B**), but not PLR (**C**) were significantly associated with worse survival (log-rank *P* < 0.05). (**D**)The patients with concomitant high-NLR & low-LMR have the worst overall survival (log-rank *P* < 0.05).

The mean absolute neutrophil, lymphocyte, monocyte and platelet counts were 4.8 ± 3.0 × 10^3^/μl, 1.4 ± 0.8 × 10^3^/μl, 0.5 ± 0.2 × 10^3^/μl, and 221.4 ± 92.1 × 10^3^/μl, respectively. The mean pre-treatment NLR, LMR and PLR values were 6.38, 3.45, and 14.6, respectively. During a mean follow-up period of 60.1 months (range 4–146 months), 17 patients died of ESCC progression, 45 patients died of second primary cancers (38 head & neck cancers; 3 gastric cancers; 2 lung cancers and 2 unknown primary cancers). The 5-year overall survival and ESCC-related survival rate were 57% and 86%, respectively (Fig. 1). Receiver operating characteristic (ROC) curve analysis to identify optimal cut-off values showed an NLR cut-off value of 2.5 (sensitivity: 0.77, specificity: 0.45; area under the curve [AUC]: 0.62), LMR cut-off value of 4 (sensitivity: 0.24, specificity: 0.6; AUC: 0.39), and PLR cut-off value of 5 (sensitivity: 0.92, specificity: 0.18; AUC: 0.62) to predict a poor overall survival of the patients (Fig. 2). Based on these cut-off values, subgroup analysis for survival was performed. Patients with a high NLR (> 2.5) or low LMR (< 4), but not PLR were significantly associated with a worse survival (*P* < 0.01, Fig. 3A-C). Furthermore, patients with concomitant high-NLR & low-LMR have the worst overall survival (Fig. 3D). A high NLR was correlated with longer tumor length, and larger tumor circumference extension (*P* < 0.05, Table 2) and tended to be associated with a higher risk of second primary cancer (*P* = 0.069). A low LMR tended to be associated with a larger tumor size (*P* = 0.06), larger circumferential extension (*P* = 0.09) and deeper cancer invasion depth (*P* = 0.09). Compared to the existing predicting system using the pathological features, including presence of deep submucosal invasion [HR: 0.74, 95% CI: 0.37–1.49, *P* = 0.402] or presence of lymphovascular invasion [HR: 1.25, 95% CI: 0.51–3.06, *P* = 0.627], the LMR < 4 [HR: 2.48, 95% CI: 1.11–5.53, *P* = 0.026] but not NLR > 2.5 [HR: 1.56, 95% CI: 0.70–3.45, *P* = 0.278] is a significant independent predictor for a worse long-term outcome after resection. Multivariate Cox regression analysis showed that presence of second primary cancers (HR: 5.05, 95% CI: 2.75–9.28, *P* < 0.001), low LMR (HR: 2.56, 95% CI: 1.09–6.03, *P* = 0.03), but not NLR (*P* = 0.75) were independent risk factors associated with a poor prognosis (Table 3).

**Table 2:** The relationships between the clinical characteristics with NLR and LMR.

Variables	NLR		*P* value	LMR		*P* value
	< 2.5 (N = 56)	> 2.5 (N = 100)		< 4 (N = 101)	> 4 (N = 55)	
Age	52.5 ± 7.8	52.0 ± 9.62	0.73	53.0 ± 9.8	50.7 ± 7.0	0.13
**Sex**						
Female	1	4	0.56	2	2	0.63
Male	55	96		99	53	
Alcohol	52	94	0.78	94	52	0.72
Betel nut	33	69	0.20	66	36	0.99
Cigarette	55	92	0.11	94	53	0.40
Tumor length, cm	3.0 ± 1.8	3.9 ± 2.9	0.045	3.9 ± 2.9	3.1 ± 1.9	0.06
Circumferential extension > 1/2	21 (37%)	57 (57%)	0.03	55 (55%)	22 (40%)	0.09
**Invasion depth**						
Mucosal	44 (79%)	71 (71%)	0.30	70 (69%)	45 (82%)	0.09
Submucosal	12 (21%)	29 (29%)		31 (31%)	10 (18%)	
Lymphovascular invasion	10 (18%)	11 (11%)	0.23	13 (13%)	8 (15%)	0.11
Second primary cancer	19 (34%)	49 (49%)	0.069	47 (47%)	21 (38%)	0.315

**Table 3:** Multivariate Cox regression analysis to determine the predictors of a poor prognosis.

Risk factors	Multivariate analysis		
	Adjusted OR	95% CI	*P* value
NLR > 2.5	1.16	0.47–2.89	0.75
LMR < 4	2.56	1.09–6.03	0.03
PLR > 5	0.84	0.28–2.89	0.85
Tumor length	1.01	0.91–1.13	0.84
Submucosal invasion cancer	0.66	0.33–1.31	0.23
Lymphovascular invasion	1.10	0.44–2.76	0.83
Second primary cancer	5.05	2.75–9.28	< 0.001

## Discussion

Esophageal cancers are a major global health issue with poor prognosis despite advances in treatment modalities in recent decades. Currently, the existing treatment guidelines suggest using the pathological features and staging in resected specimes to predict the long-term outcome of superficial ESCC^14,15^. There are no pre-treatment non-invasive biomarkers available to stratify the risk of a poor prognosis. In the present study, we assessed the roles of NLR, LMR and PLR in SESCC, and identified that a low LMR (< 4) was an effective predictor of poor survival. The predictive performance of LMR is better than the currently existing system, potentially because it may reflect the balance between pro-cancer inflammatory and anti-cancer immune responses, as well as the potential impacts on developing second primary cancers that may reduce the overall survival. In contrast, the pathological findings in resected specimens just reflect the local status. To the best of our knowledge, the present study is the first to assess the roles of these inflammatory markers in patients with early-stage esophageal cancers. The pre-treatment value of LMR is easily obtainable, and in the age of individualized patient care and precision medicine, it may represent a risk stratification tool for superficial ESCC patients.

Previous studies have shown that inflammation plays a key role in cancer development, treatment effect, and long-term surviva^17,26^. The proposed mechanism is that cancer-related inflammation can cause DNA damage, promote angiogenesis and cell proliferation, suppress antitumor immunity, and impact the response to anticancer therapies^27,28^. In addition, inflammatory responses may help to induce certain populations of tumor stem cells which are critical for resistance and metastasis of tumor tissue^29^. Tumor-infiltrating neutrophils may be associated with a poor prognosis through the promotion of angiogenesis, cell mobility, and migration^17^. Lymphocytes have been associated with tumor cell removal and improved tumor surveillance. Previous studies have demonstrated an association between a low peripheral lymphocyte count and poorer survival in different types of cancer^30,31^. Activated platelets can induce coagulation and interact with tumor cells through paracrine signaling or direct contact, thereby inducing tumor cell growth, angiogenesis and poor survival^32,33^. Monocytes, which differentiate into tissue macrophages and dendritic cells, can mediate tumor-associated monocyte infiltration in solid tumors, and induce various chemokines such as transforming growth factor (TGF)-α, tumor necrosis factor (TNF)-α, interleukin (IL)-1, and IL-6 to promote tumorigenesis, angiogenesis and distant metastasis^34,35^. This may explain the association between a high monocyte count and poorer survival. NLR, LMR, and PLR have been reported to predict the potential suppression of host immune response and survival of cancer patients^16,17^. One study demonstrated that patients with ESCC and an NLR > 2.33 had a better pathological response after neoadjuvant chemoradiotherapy^22^. A recent study also found that high NLR (> 2.20) and PLR (> 110) were associated with larger tumor size, and that NLR was a prognostic factor for resectable ESCC^23^. Another retrospective study of 1587 ESCC patients who underwent esophagectomy found that a high pre-operative NLR (> 3.29) and low LMR (< 2.95) could predict worse survival in patients with ESCC^24^. A recent meta-analysis also confirmed that a high NLR may predict a poor prognosis, including overall survival, cancer-specific survival, and progression-free survival of ESCC patients^36^. However, the roles of NLR, LMR, and PLR in patients with early-stage cancers have rarely been reported. In the present study, we found that the patients with a high pretreatment NLR (> 2.5) or low LMR (< 4) had worse overall survival, suggesting that these parameters could serve as non-invasive, easily obtainable markers in clinical practice. In addition, we found that the patients with a high NLR or low LMR tended to have a larger tumor longitudinal and circumferential extension, suggesting that a lower lymphocyte count and relatively weak anti-tumor immunity may predispose to a larger tumor size and poor long-term survival. Moreover, due to the field cancerization theory^9^, the risk of developing second primary cancers were very high (43.5%) in SESCC patients and these may lead to a poor prognosis. Thus, surveillance for second primary is becoming an important issue for patients with SESCC. Our study showed patients with high-NLR tended to have second primary cancers (*P* = 0.069). The presence of second primary cancer or low-LMR were the significant independent predictors for worse survival. These findings not only provide a potential biomarker to predict the prognosis of superficial ESCC, but also provide a clue for the future development of immunotherapy.

There were several limitations to the current study. First, we did not investigate the effect of sequential changes in LMR on recurrent events and treatment response. Second, this study was conducted at a single medical center, and further multicenter studies are required to validate the performance of LMR as a risk assessment marker for SESCC. In conclusion, the patients with SECC (clinical Tis or T1N0M0) and a low pre-treatment LMR in peripheral blood in this study had a poorer prognosis. LMR values can be easily obtained from routinely collected blood samples, and could assist clinicians when deciding the surveillance program for SESCC patients.

In conclusion, minimally invasive endoscopic resection or esophagectomy is the standard treatment for SESCC. However, some patients may occur disease progression or develop second primary cancers after the therapies. Our study identified a low pre-treatment LMR may be a non-invasive pre-treatment predictor of poor prognosis to guide the surveillance program of SESCC, suggesting that anti-cancer immunity may play a role in the early events of esophageal squamous cancer.

## Data Availability

The datasets used and/or analysed during the current study available from the corresponding author on reasonable request.
